# Evaluation of the antimicrobial effects of MTAD, NaOCl against selected endodontic pathogens

**Published:** 2009-04-17

**Authors:** Mohammad Asna Ashari, Fariba Fayaz, Nahid Moezzi Ghadim, Laleh Alim Marvasti, Yadollah Mehrabi

**Affiliations:** 1 *Department of Endodontics, Iranian Center for Endodontic Research, Dental School, Shahid Beheshti University of Medical Sciences, Tehran, Iran.*; 2 *Department of Microbiology, Shahid Beheshti University of Medical Sciences, Tehran, Iran.*; 3 *Dentist, Private Practice, Tehran, Iran.*; 4 *Dentist, Private Practice, EDO Department, Dental School, Shahid Beheshti University of Medical Sciences, Tehran, Iran.*; 5 *Shahid Beheshti University of Medical Sciences, Tehran, Iran.*

**Keywords:** *Candida albicans*, *Enteric bacteria*, *Enterococcus faecalis*, MTAD, Sodium hypochlorite, *Staphylococcus aureus*

## Abstract

**INTRODUCTION:** The aim of this study was to compare the antimicrobial effects of MTAD, sodium hypochlorite (NaOCl) and their combination on endodontic micro-organisms.

**MATERIALS AND METHODS:** Zone of Inhibition (ZI) and Minimum Inhibitory Concentration (MIC) were the techniques used. In ZI technique blood agar plates were inoculated with organisms, paper discs were soaked with irrigants and maximum zones of bacterial inhibition were recorded. In the MIC technique the irrigants were serially diluted in TSB tubes and 0.1 mL of the tested microbe solutions were added. Results were obtained on the basis of turbidity and growth on agar plates. Statistical analyses were carried out using ANOVA and Tukey tests.

**RESULTS:** In ZI technique, we investigated 120 specimens including 5 microbial species, 3 irrigants and their control groups, each with 6 repetitions. The results demonstrated MTAD greater antimicrobial efficacy compared to NaOCl, and their mixture (M+N) against *Staphylococcus *(*S*)* aureus*, *Enteric *(*E*)* bacteria* and *Enterococcus *(*E*)* faecalis* (P<0.001). NaOCl was more effective in eradicating *Candida *(*C*)* albicans* than the others (P<0.01). MIC method (155 tubes) showed MTAD to be more effective against *E. bacteria* and *S. aureus*. MTAD and NaOCl were equally effective against *E. faecalis*; however, NaOCl was more effective against *C. albicans*.

**CONCLUSION:** Bacterial species were more susceptible to MTAD than NaOCl, *C. albicans*, however, was more susceptible to NaOCl. The advantage of NaOCl is that it has broad spectrum antimicrobial activity. The joint solution (M+N) did not prove to be more effective than their individual use.

## INTRODUCTION

It is well established that pulp and periapical disease as well as failed root canal therapy (RCT) are due to the presence of microbes in the root canal system (1). Eliminating microbes from the infected root canals and prevention of re-infection are one of the fundemental aims of RCT. Clinical investigations have shown that chemo-mechanical preparation with the use of anti microbial medicaments will effectively reduce the microbial content of the canals (2-4). Despite these meticulous efforts some micro-organisms stay within the canal (4-7). In a clinical study evaluating the effect of infection on the RCT outcome, Sjogren *et al*. cultured samples from the root canals after preparation and before obturation. When the cultures were negative, the success rate was 94%; when bacteria were found however, success rate was reduced to 68% after 5 year follow up (5).

Extensive research has discovered various microorganisms in root canal failures (Chronic Apical Periodontitis) such as *Enterococci* (8-15), *Actinomyces* (13), *Streptococci* (13), *Enterobacter* (11,12,16,17), *Fungi* (14,17-21) and *Staphylococci* (S) (11,12,15). *Enterococcus* (E) faecalis, the most prominent, is present in 30-70% of failure cases (10,14,17,22,23). *Enteric (E) bacteria* form approximately 5% (16) and *fungi* 7% (24) of the infected root canal micro flora.

Various Sodium Hypochlorite (NaOCl) concentrations have been used over the years as an irrigant in RCT. The main benefits of NaOCl are its ability to dissolve necrotic tissue (25), its broad spectrum antibacterial action (26,27) and lubricating effect during filing and irrigation of debris within the canals (28). However unpleasant taste and odor, toxicity (29), resorption (30), inability to remove smear layer and fully eradicate microbes from the infected canals (5,9,31) are the main disadvantages of this popular irrigant. To remove the remained bacteria, medicaments can be used between treatment sessions though their effectiveness in eradicating some microbes such as *E. faecalis and Candida (C) albicans* is questionable (4,17,32-35).

Recently a new irrigant called MTAD (a mixture of tetracycline isomer, an acid and a detergent) has been introduced as a final irrigant to be used after NaOCl for disinfecting the root canal into the market (36). The results of some investigations have shown that MTAD can effectively remove the smear layer and abolish E. *faecalis* (37-40) though some researchers are still adamant that NaOCl is more effective (41-44).

We decided to compare the antimicrobial effect of NaOCl, MTAD, and their mixture against resistant microbes in RCT with Zone of Inhibition (ZI) and Minimum Inhibitory Concentration (MIC) *in-vitro*.

## MATERIALS AND METHODS

The investigation was carried out in Shahid Beheshti University Dental School and approved by the ethical committee. The microbes tested were *E. faecalis*, *Pseudomonas *(*P*) *aeruginosa*, *Escherichia *(*E*) *coli*, *S. aureus* and *C. albicans*. *E. faecalis* was incubated in Bile Scoline Agar, *P. aeruginosa* and *S. aureus* in Blood Agar, *E. coli* in Esoin Methylen Blue (EMB), and *C. albicans *in Sabouraud Dextrose Agar plates. After 24 hours a suspension was prepared from each microbial species with a concentration of 1 McFarland (*i.e.* 3×10^8^ Colony of bacteria in the Trypticase Soy Broth environment) and used in the investigation.

To assess the antimicrobial power of each irrigant ZI and MIC was employed. In the ZI technique 30 blood agar plates (batch no: 108860500, Merck KGaA, Darmstadt, Germany) were prepared according to manufacture’s instructions and the 125 mm Petri dishes were uniformly covered. These were then stored for 2 days in 37^°^C to make sure sterilized conditions were met and bacteria were not introduced. The sterile paper discs (batch no: 1288502, Padtan Teb, Tehran, Iran) diameter of 6 mm were divided into three groups. Each group was soaked either with 4 drops (0.2 mL) of NaOCl, MTAD or an equal mixture of MTAD and NaOCl (M+N).

Five mL from each 1 McFarland suspension was removed with a sterile swab next to a flame and under the ventilation hood and placed on a blood agar plate in a criss-cross fashion. Each culture area was then divided into 4 sections in the following manner: the first section contained disc soaked with BioPure MTAD (batch no: 050805, Dentsply Tulsa Dental, Tulsa, OK), the second section with NaOCl 5.25% (batch no: 28095, Pure Sodium Hypochlorite, Chemeen Company, Iran) and the third section contained disc soaked with equal amounts of MTAD and NaOCl (5.25%), the final section consisted of normal saline (batch no: 173885 A, 0.9% Sodium Chloride, Medicines and Drugs Company, Iran). This was repeated 6 times for each of the microbes; altogether 120 samples were taken. Samples were placed in the incubator (batch no: 0323212100, Farateb Tajheez Specialist Company, Iran) for 48 hours at 37^°^C and 10% CO_2_. The maximum microbial ZI around each disc was then measured and recorded in millimetres.

In the MIC technique 30 test tubes were prepared for each microbe. One mL of Trypticase Soy Broth (TSB) (batch no: 105459, Merck KgaA, Darmstadt, Germany) was poured into each test tube and then autoclaved (sterilization). The tubes were then divided into three groups of 10 test tubes each. One mL of the selected irrigant was placed in the first test tube of each group and shaken with Cenco instrument MIJ (batch no: 5444, Holland) to homogenise the fluid. Subsequently, 1 mL of the solution was taken from the first test tube and transferred into the next tube; this was then repeated for all the ten tubes. The irrigant was gradually diluted from 1:2 to 1:1024 in the last test tube. An extra test tube was used with normal saline as the control. Exactly 0.1 mL of the selected microbial suspension (1 McFarland) was added to each of the test tubes, and this was performed for all the tubes and investigated irrigants. Test tubes were placed in the incubator for 24 hours (37^°^C and 10% CO_2_).

**Table 1:** Statistics of inhibition zones and all the 5 microorganisms tested

Groups	N	Mean	SD
***Enterococcus Faecalis***	18	22.111	5.189
***Escherichia Coli***	18	21.500	3.535
***Pseudomonas aeroginosa***	18	22.722	5.210
***Candida albicans***	18	25.555	19.281
***Staphylococcus aureus***	18	33.277	5.798
**Total**	90	25.033	10.454

**Table 2:** Statistics of inhibition zones and the 3 irrigants for 4 microorganisms tested

Irrigants	N	Mean	SD
**MTAD**	24	28.500	6.036
**NaOCl**	24	25.916	5.740
**MTAD/NaOCl**	24	20.291	6.457
**Total**	72	24.902	6.920

**Table 3:** Statistics of inhibition zones and the 3 irrigants for 5 microorganisms

Irrigants	N	Mean	SD
**MTAD**	30	22.800	12.780
**NaOCl**	30	29.366	8.825
**MTAD/NaOCl**	30	22.933	8.064
**Total**	90	25.033	10.454

**Table 4:** MIC test of the 5 different microorganisms

Microorganism	MTAD	NaOCl	MTAD/NaOCl
***E. faecalis***	1:32	1:32	1:8
***P. aeroginosa***	1:64	1:8	1:8
***E. coli***	1:128	1:32	1:32
***S. aureus***	1:512	1:32	1:16
***C. albicans***	1:4	1:16	1:8

**Figure 1:**
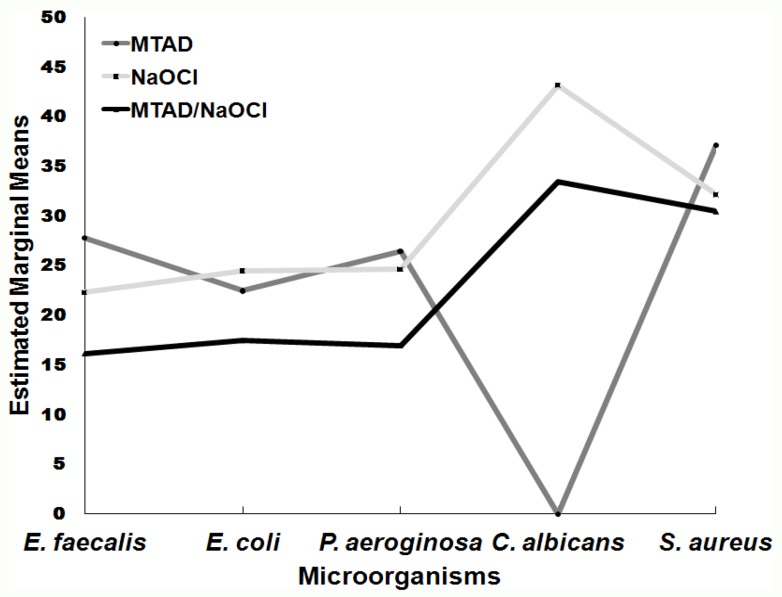
Estimated Marginal means of inhibition zones for the 5 bacteria

MTAD and MTAD+NaOCl were partially opaque and therefore a culturing method was also employed. After measuring turbidity of the test tubes, 0.5 cc of each tube solution was placed on Nutrient Agar (batch no: 1054430500, Merck KGaA, Darmstadt, Germany) and placed in the incubator for 48 hours. One hundred and fifty five test tubes were used to test the 5 individual microbes, 5 of which were controls. The MIC of the irrigants for each individual microbe was recorded based on the degree of turbidity (with the naked eye) and the absence of microbial growth on the agar plates. The experiment was carried out in accordance with sterilisation principles (45).

The relevant results of ZI were added into SPSS for Windows V 11.5. Descriptive criteria (central values, measurements of dispersion and confidence interval levels) were calculated and the data was analysed with ANOVA and Tukey tests and P<0.05 was considered statistically significant.

## RESULTS

The results for the ZI technique displayed that *S. aureus* was statistically the most sensitive micro-organism (maximum ZI) against investigated irrigants (P<0.002) (Table 1).

MTAD was more effective than 5.25% NaOCl and NaOCl was more effective than MTAD+NaOCl against *E. faecalis*, *S. aureus* and *E. bacteria* (P<0.001) (Table 2).

Overall NaOCl showed statistically greater antimicrobial power against *E. faecalis*, *S. aureus*, *E. bacteria* and *C. albicans*: NaOCl>MTAD=MTAD+NaOCl (P<0.019) (Table 3 and Figure 1).

*C. albicans* was the most resistant micro-organism to MTAD (P<0.001).

MIC results illustrated that MTAD and NaOCl were strong antimicrobials and may be equally effective even when diluted. In this study MTAD was more effective against *S. aureus *and *E. bacteria*, however, NaOCl 5.25% and MTAD were equally effective against *E. faecalis* (Table 4).

## DISCUSSION

The results of this *in vitro* investigation reiterate the superiority of MTAD compared to NaOCl and M+N against *S. aureus*, *E. bacteria* and *E. faecalis* by the ZI method. The poorest antimicrobial agent was shown to be the mixture of M+N.

Experiments carried out by Davis *et al. *(40) and Krause *et al. *(43) in 2007 both proved MTAD to be more effective against *E. faecalis* than NaOCl 5.25%. This study also demonstrates that MTAD is effective against a range of bacteria.

Tay *et al.* carried out a similar study that compared MTAD, NaOCl and the consecutive use of the irrigants (instead of combination) (46). They used a concentration of 1.3% NaOCl; their results confirmed that MTAD was the most effective irrigant in eliminating *E. faecalis*. However, they found no difference between NaOCl and N+M; possibly due to the different concentration of NaOCl, bacterial species and/or slightly different incubation conditions employed.

Torabinejad *et al.* has compared the effectiveness of MTAD and NaOCl (5.25%) using the ZI technique and discovered their similar antibacterial action against *E. faecalis *(38). However, in this study after dilution MTAD was shown to have a significantly greater ZI than NaOCl.

Interestingly the antimicrobial effect of the mixture (M+N) was less than MTAD or NaOCl alone. A recent study showed that MTAD used alone has greater substantivity than other irrigants (47). NaOCl reduces the antimicrobial power of MTAD; both irrigants are stronger antimicrobials when used independently. Attention must be paid to the oxidation of MTAD by NaOCl which reduces the substantivity and antimicrobial power of MTAD; similar to the peroxidation of tetracycline with Reactive Oxygen Species (ROS), confirmed in Tay’s study (46).

There have been reports that antioxidant such as ascorbic acid rinse following NaOCl irrigation will remove remnants of hypochlorite (48).

*C. albicans* distorts the findings as NaOCl is stronger agent than MTAD and M+N; MTAD showed no antifungal effects. Ruff *et al’s* study also supports our results; they demonstrated that Chlorohexidine 2% and NaOCl 6% were most effective in reducing the CFU of *C. albicans* (49).

There is a large body of evidence including this current study that find bacteria such as *E. faecalis*, strands of *E. bacteria* (*P. aeruginosa*, *E. coli*) and types of *C. albicans* to have greater resistance to irrigation when compared to *S. aureus* (8-21). These microorganisms have also been known to be resistant to antibacterials and intracanal medicaments such as calcium hydroxide (50-52).

MIC results illustrated that MTAD and NaOCl were strong antimicrobials even after dilution. In this study, MTAD was more effective against *S. aureus* and *E. bacteria*, thought NaOCl 5.25% and MTAD were equally effective against *E. faecalis*.

A similar study using MIC, Torabinejad *et al.* demonstrated that MTAD was effective when diluted 200 times, and NaOCl (5.25%) was effective when diluted 32 times against *E. faecalis* (38), concurring with our results (MTAD was found effective when diluted 32 times). This may be due to the various strands of *E. faecalis*, different amounts of doxycycline in MTAD (43), conditions of incubation used.

The present study used Bio Pure MTAD (Dentsply, Tulsa Dental, Tulsa, OK) with 150 mg of Doxycycline i.e. 3%. MTAD is naturally opaque and therefore measuring its turbidity may cause discrepancy in the results. MTAD instructions require NaOCl to be used as the final irrigant (36). The results in this study and Tay *et al’s* indicate that an interaction may occur altering the antimicrobial efficacy of MTAD (46). The advocacy of using these irrigants consequetively may be questioned.

We suggest further investigations using irrigants in a polymicrobial environment to mirror endodontics infections.

## CONCLUSION

MTAD is ineffective against *C. albicans* and its substantivity may be altered when used in conjunction with NaOCl. *C. albicans* is often present in resistant and secondary endodontic infections as well as in peri-radicular lesions. NaOCl is an inexpensive and readily available chemical with broad spectrum action which has stood the test of time.
